# Dual-action benefits: global (action-inherent) and local (transient) sources of action prepotency underlying inhibition failures in multiple action control

**DOI:** 10.1007/s00426-022-01672-0

**Published:** 2022-04-08

**Authors:** Jens Kürten, Tim Raettig, Julian Gutzeit, Lynn Huestegge

**Affiliations:** Department of Psychology, Institute of Psychology, University of Würzburg, Röntgenring 11, 97070 Würzburg, Germany

## Abstract

Previous research has shown that the simultaneous execution of two actions (instead of only one) is not necessarily more difficult but can actually be easier (less error-prone), in particular when executing one action requires the simultaneous inhibition of another action. Corresponding inhibitory demands are particularly challenging when the to-be-inhibited action is highly prepotent (i.e., characterized by a strong urge to be executed). Here, we study a range of important potential sources of such prepotency. Building on a previously established paradigm to elicit dual-action benefits, participants responded to stimuli with single actions (either manual button press or saccade) or dual actions (button press and saccade). Crucially, we compared blocks in which these response demands were randomly intermixed (mixed blocks) with pure blocks involving only one type of response demand. The results highlight the impact of global (action-inherent) sources of action prepotency, as reflected in more pronounced inhibitory failures in saccade vs. manual control, but also more local (transient) sources of influence, as reflected in a greater probability of inhibition failures following trials that required the to-be-inhibited type of action. In addition, sequential analyses revealed that inhibitory control (including its failure) is exerted at the level of response modality representations, not at the level of fully specified response representations. In sum, the study highlights important preconditions and mechanisms underlying the observation of dual-action benefits.

## Introduction

Performing many actions at the same time can be challenging. This intuitive notion is reflected in a large body of corresponding research on multiple action control and multitasking. Performance (in terms of response speed and accuracy) is usually worse in conditions requiring the execution of two actions compared to conditions with only a single-action requirement (e.g., Pashler, 1994). Such dual-action costs*,* however, do not always have to be the price paid for multiple overt action execution. Recent evidence demonstrates that in some situations, executing two actions can actually result in better overall performance than executing one action while withholding the other (Miller, 2006). Such dual action benefits can occur when a particular action is sufficiently prepotent to require active inhibition in single-action trials when only the alternative response should be executed. Sources of action prepotency can be broadly categorized into those which are temporally stable and action-inherent (or global), such as the overall ease of execution of a particular action, and those which are more transient (or local), as reflected in trial-by-trial effects of switching between response demands. The current study seeks to determine the relative impact of both types of sources in a dual-action setting involving oculomotor responses (i.e., saccades) and manual (button press) responses.

### Accuracy-related dual action benefits and action prepotency

Being involved in two types of action at the same time can sometimes be easier than focusing on one action while trying to suppress another. For example, it has been suggested that performing mental arithmetic tasks was easier while being allowed to move at the same time as compared to being required to sit still (Langhanns & Müller, 2018). In a more basic study that first explicitly focused on the exact conditions underlying the occurrence of performance benefits associated with dual-action execution, participants responded to a single peripheral stimulus in a spatially compatible manner with either a single eye movement (saccade), a single manual button press, or with both concurrently (Huestegge & Koch, 2014). Importantly, single-action demands were accompanied by the explicit instruction not to execute the alternative action. In single manual trials, for example, participants were instructed to remain fixated and not make a saccade toward the target. The main result was the observation of a large proportion of erroneously executed saccades (i.e., false positives) in trials requiring only a manual button press, compared to few errors in trials affording both actions. The same pattern of results has been replicated when using manual and vocal responses, thereby demonstrating that this phenomenon is not restricted to the special case of oculomotor control (Raettig & Huestegge, 2018, 2021): When required to only respond vocally to a central visual stimulus, participants executed a substantial amount of unwarranted (i.e., false-positive) manual responses in a situation with frequent switches between responds demands (single, dual). These errors represent instances in which the demand to execute only one action while inhibiting another proved to be difficult to the extent that single-action performance costs (or, conversely, dual-action benefits) in response accuracy were observed. Note that in some conditions (and participants) of these experiments, such dual-action benefits in error rates (ERs) went hand in hand with dual-action costs in reaction times (RTs). While it is difficult in these cases to draw final conclusions regarding overall performance, such observations do not endanger the interpretation of dual-action benefits in relation to inhibitory control failures, which are solely defined with regard to performance accuracy.

In these previous studies, potentially important theoretical preconditions were derived for observing this type of dual-action benefits. In particular, the presence of dimensionally overlapping (both containing a spatial component) and compatible (both left or both right) responses (Kornblum et al., 1990) as well as unpredictability regarding the particular responses and response types from trial to trial may represent prerequisites of inhibition failures in single-action trials. On a theoretical level, inhibitory failures could be due to spreading activation from an activated spatial code (e.g., “left”) required in a given trial to a closely associated but currently unrequired modality code (e.g., saccade), eventually triggering an erroneous response in this modality. Such a mechanism would largely depend on a high pre-activation (or prepotency) of the respective response modality, such as saccades in the context of additional manual responses (Huestegge & Koch, 2014), or manual responses in the context of additional vocal responses (Raettig & Huestegge, 2018, 2021).

A similar mechanism based on inhibitory costs for highly prepotent responses was also proposed to explain a related phenomenon, namely the no-go backward crosstalk effect (no-go BCE; Janczyk & Huestegge, 2017; Miller, 2006). In a typical dual-task paradigm combining a choice response Task 1 with a go-/no-go Task 2, Task 1 performance can be adversely affected by a no-go demand in Task 2 when the go response is highly prepotent and thus difficult to inhibit.

The notion of action prepotency refers to the idea that the easier an action is prepared and initiated in a given context, the harder it becomes to suppress it (Ridderinkhof et al., 2014). Applying this idea to the domain of dual-action benefits, this implies that dual-action benefits can principally emerge based on inhibitory demands in any response modality (e.g., saccades, manual responses), given sufficient action prepotency in a particular trial.

In the study by Huestegge and Koch (2014), false-positive saccades were substantially more frequent than false-positive manual responses pointing toward higher prepotency of the saccade response (compared to the manual response). Saccade prepotency was additionally increased by implementing a “gap condition” (Saslow, 1967), in which the central fixation cross disappeared 200 ms prior to onset of the imperative stimulus in the periphery, in addition to a baseline fixation condition in which the fixation cross remained present throughout (overlap condition, see Huestegge and Koch, 2010b). Accordingly, the proportion of false-positive saccades in single manual trials was slightly higher in the gap (vs. overlap) condition, demonstrating that saccade prepotency in terms of the overall ease of execution (Forbes & Klein, 1996; Jin & Reeves, 2009; Ross & Ross, 1980) was indeed directly linked to saccade inhibition difficulties.

However, action prepotency in a given trial should not only be determined by global (action-inherent) factors (such as a greater “urge” to execute saccades to peripherally occurring targets as opposed to manual key press actions), but also by more local (transient) trial-by-trial effects in working memory that are, for example, driven by after-effects of previous trials or anticipations of future trial demands. While trial-by-trial effects on motor actions are well documented in various psychological domains (e.g., Campbell & Proctor, 1993; Pashler & Baylis, 1991), their role in determining action prepotency as a factor underlying accuracy-based dual-action benefits has remained unexplored yet.

### Local (transient) changes in action prepotency

In previous dual-action benefit studies conducted by Huestegge and Koch (2014) and Raettig and Huestegge (2018, 2021), single-action and dual-action trials were always intermixed within blocks. Such a single-dual switch paradigm (SDS paradigm, e.g., Huestegge & Strobach, 2021; Strobach & Huestegge, 2021) was deemed particularly suited to reveal inhibition-based dual-action benefits as inhibition of an action should be especially difficult when the same action had to be overtly executed in the preceding trial—and may potentially have to be executed on the following trial.

This reasoning is based on research showing that when switching between task conditions with varying action demands (e.g., single, dual), contents of previous and anticipated subsequent trials can have a major impact on performance in the current trial (for a review on corresponding effects in task switching, see Kiesel et al., 2010). These effects have been traditionally explained in terms of sub-optimal advance task set (re-)configuration (Rogers & Monsell, 1995) or persisting activation (or inhibition) of actions recently executed (or suppressed). More recent accounts refer to the retrieval of previously established event files (i.e., composites of task feature representations relevant in a given trial, e.g., stimuli, responses) due to repetition of features shared with previous trials (e.g., Frings et al., 2020). Essentially, persisting past events as well as (the anticipation of) future events can cause crosstalk within the ongoing trial (Navon & Miller, 1987) that can eventually lead to performance benefits or costs, depending on the particular circumstances.

**Fig. 1:**
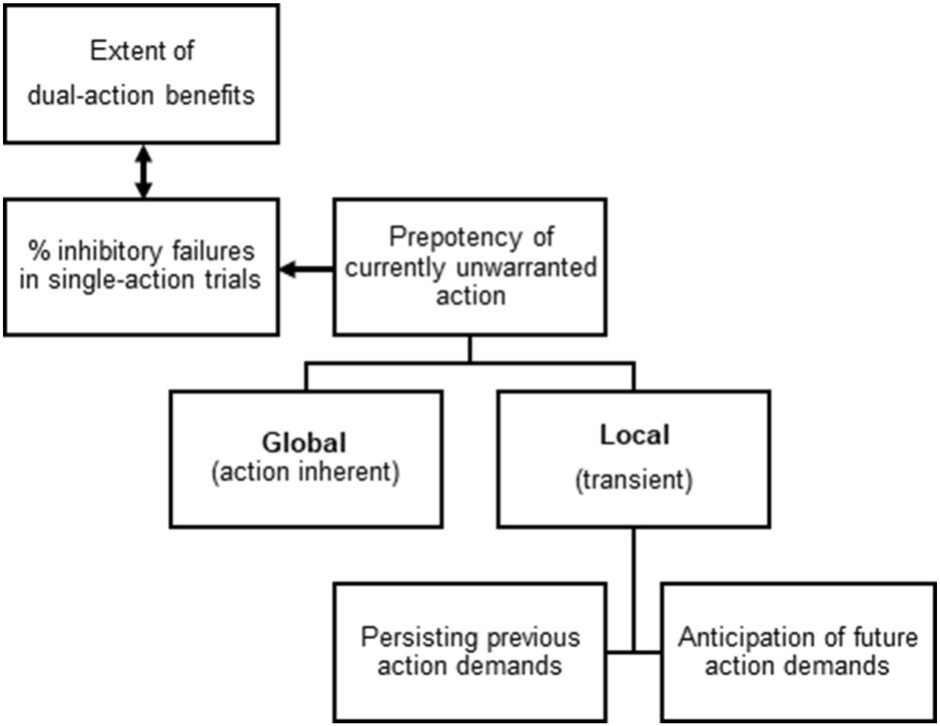
Determinants of action prepotency as an underlying factor affecting the extent of dual-action benefits. The presence and strength of dual-action benefits is determined by the frequency of inhibition failures in single-action trials (in relation to errors in dual-action trials). The frequency of inhibition failures depends on the prepotency of the currently unwarranted action in single-action trials. Prepotency is (among others) determined by global (action-inherent) factors (e.g., the overall ease or urge to execute a particular action in general, e.g., a saccade) and on local (transient) changes due to persisting activation of previous response demands or the anticipation of future response demands

Transferring these ideas to the context of dual-action benefits, this suggests that persisting previous action demands as well as anticipations of future action demands (e.g., execution of a saccade) could have a major (adverse) impact on action control (in terms of higher unwarranted action prepotency) in the current trial (e.g., execution of a button press in the absence of saccade execution). Most interestingly, though, an analysis of corresponding sequential effects that may indicate persisting activation from the previous trial in the study by Huestegge and Koch (2014) revealed that the occurrence of false-positive saccades in single manual trials was not clearly contingent upon the requirement to perform a saccade in the previous trial (probably in part due to the fact that this previous study was not explicitly designed to focus on such sequential effects). The role of anticipations of future action demands, on the other hand, could not be explicitly addressed, since in intermixed designs such expectancies should be present throughout. This highlights the importance of investigating how the need to switch between single-action and dual-action demands from trial to trial affects action prepotency when compared with a control condition without such switches. This would allow us to explicitly assess the role of local prepotency effects in observing dual-action benefits in a systematic fashion (see Fig. 1).

### The present study

The present study builds on the previous experiment conducted by Huestegge and Koch (2014) and combines oculomotor and manual responses required in the context of single-action and dual-action trials. Crucially, however, we added a manipulation of response demand context by having participants experience blocks with switching, unpredictable response demands (mixed blocks: single AND dual actions) and blocks with constant, predictable response demands (pure blocks: EITHER single OR dual actions). A constant response demand context should minimize the influence of preceding or subsequent trial types (tapping into persistence- or anticipation-based effects, respectively) on action prepotency in a current trial. Unlike all previous studies, this allowed us to precisely assess the impact of global (action-inherent) as opposed to local (transient) sources of action prepotency on dual-action benefits.

Specifically, participants responded to a single peripheral visual stimulus with either a single manual button press, a single saccade, or both. Using only one stimulus to prompt responses (including dual actions), we reduced any potential for additional difficulties regarding input-related or centrally based (i.e., due to two conflicting response selection processes) interference, instead focusing only on action-related control processes of a single compound response selection (Fagot & Pashler, 1992).

The most critical dependent variable indicating inhibition failures related to dual-action benefits is the proportion of false-positive errors in single-action trials (i.e., saccades executed in single manual trials; button presses executed in single saccade trials) compared to errors of any other kind (i.e., false-negative errors, directional errors) in dual-action trials. Based on previous findings, we expected dual-action benefits to be most pronounced in erroneous saccade responses (as saccades to peripherally presented targets were assumed to exhibit high global (action-inherent) execution prepotency), but—as outlined above—inhibition failures are also reasonable to expect in manual responses. Given the lower working memory load and better predictability of task demands in pure blocks, overall task performance should be better in these blocks than in mixed blocks (similar to mixing costs, see Koch et al., 2018). However, the exact pattern of results (i.e., the specific differences in false-positive error rates between conditions) can provide important insight into the effect of demand context on inhibitory control failure underlying dual-action benefits. Based on different theoretical assumptions, four potential outcomes were principally deemed reasonable:

(1) If a global (action-inherent) action prepotency is the main determinant of (e.g., oculomotor) inhibition failures (e.g., because saccade responses are generally prepotent, irrespective of any prior or successive response demands), one would expect to find substantial dual-action benefits even in pure blocks. If such a global (action-inherent) prepotency represents the sole causal factor behind inhibitory failures, one would even expect the frequency of false-positive responses in pure blocks to be on par with that in mixed blocks. In addition, based on this account one could expect a main effect of fixation condition (gap, overlap), as gap conditions might further boost the already high level of oculomotor (compared to manual) action prepotency. (2) If, conversely, the frequent inability to inhibit unrequired responses in single-action trials is mainly based on local (transient) changes in action prepotency in a mixed response demand context (e.g., execution of false-positive saccades either due to a saccade demand in the previous trial or due to the anticipation of saccade demands in impending trials), no (or negligible) dual-action benefits (neither in gap nor in overlap saccade conditions) should emerge in a constant response demand context (pure blocks). Accordingly, the rate of false-positive errors in single-action trials should drop to a negligible level in pure blocks compared to mixed blocks. (3) Any result pattern in between these two extreme predictions would of course indicate that global (action-inherent) and local (transient) sources of action prepotency both play a role in determining inhibition difficulty. Such an account would still predict a greater amount of inhibition failures in saccades (vs. manual button presses), and potentially a further slight increase in false-positive saccades in gap (vs. overlap) conditions. Importantly, this account would additionally predict more frequent inhibition failures in mixed (vs. pure) blocks due to local changes in action prepotency. (4) Finally, in relation to the exact nature of any local effects on action prepotency (if present), a strong effect of block type (pure vs. mixed) on false-positive rates in the absence of substantial trial-by-trial effects would suggest that these local effects are mainly driven by anticipation of future competing response demands (instead of persisting activation, see Fig. 1). In contrast, strong trial-by-trial effects of a size that is comparable to that of the block type effect would suggest that persisting activation, not anticipation, plays a major role.

As an additional exploratory analysis, we will also assess whether persistence-based local effects (if present) are driven by, for example, persisting activation of a fully specified previous response (e.g., failure to inhibit a rightward saccade only after the requirement to execute a rightward saccade in the previous trial) or rather by persisting activation of the mere response modality active in the previous trial (e.g., failure to inhibit a rightward saccade after the requirement to execute a saccade in the previous trial, regardless of its direction).

## Methods

### Participants

Forty-eight (79% female, 94% right-handed) volunteers (mean age = 24 years, SD = 3.2 years) took part in the experiment in exchange for monetary compensation. All gave informed consent and had normal or corrected-to-normal vision without color blindness. In accord with preregistered criteria, datasets of eight participants were excluded due to an insufficient number of valid trials (< 10) in at least one of the experimental conditions. To ensure full counterbalancing, data from 8 new participants were collected. The preregistration is publicly available at osf.io/swjm7.

### Apparatus and stimuli

Participants were seated about 67 cm in front of a 21-inch CRT screen with a spatial resolution of 1024 × 768 pixels and a temporal resolution of 100 Hz. Head movements were minimized using a chinrest with forehead support. Saccade latencies and directions were registered using a desktop mounted Eyelink 1000 eye tracking system (SR Research Mississauga, Ontario, Canada) with a sampling rate of 1000 Hz.

For manual responses, participants operated two buttons (one for left responses, one for right responses) on a button box with the thumbs of their corresponding hands. A plus sign (size = 0.35° of visual angle) served as fixation stimulus at the screen center. It was initially presented in white and then changed its color to either red, green, or blue (indicating response demand, see below). The imperative stimulus was a white square (0.35° × 0.35°), which was presented at an eccentricity of 8° either to the left or to the right of the screen center. The experiment was programmed using Experiment Builder software (version 2.1.140, SR Research).

### Procedure

The experiment was conducted on two consecutive days. Before each of the two sessions, the experimenter gave verbal instructions in addition to the presentation of written instructions (see below). Speed and accuracy were equally emphasized for every required response. Participants completed six mixed blocks on one day and six pure blocks on the other day. The experiment contained a total of 720 trials (60 randomly presented trials per block) yielding 60 trials per experimental condition (30 per condition and direction). Separate days were chosen to minimize potential carry-over effects in relation to the block type manipulation. Block order was counterbalanced across participants. The first three blocks of each day involved one fixation condition (e.g., gap), and the remaining three involved the other fixation condition (e.g., overlap). Fixation condition order was also counterbalanced across participants. The entire duration of the experiment was approximately two hours (one per day). Each block started with an instruction screen displaying general task instructions followed by a nine-point calibration routine and the specific task requirements in the current block (e.g., pure: single manual).

Every trial began with the presentation of the fixation cross in white on black background for a duration of 2000 ms, after which it changed its color to either red, green, or blue. In mixed blocks, response demand varied randomly from trial to trial, and the color served as a cue to the specific response requirement (e.g., red: single saccade response, green: single manual response, blue: dual response). The mapping of colors to response demands was counterbalanced across participants using a Latin square method. In pure blocks, response demand remained constant for the entire block, thereby rendering the cue color irrelevant. The color changes were retained for purposes of comparability. 700 ms after the color change, the imperative stimulus (a white square, 0.35° × 0.35°) appeared either to the left or to the right of the screen center at an eccentricity of 8° and remained present for 2500 ms. Participants were instructed to respond in a spatially congruent manner with either a saccade toward the stimulus without pressing a button (trial type: single saccade response demand), with a spatially congruent button press while remaining fixated at the screen center (trial type: single manual response demand), or with both a saccade and a button press (trial type: dual-response demand).

To manipulate local (transient) action prepotency, mixed and pure blocks were introduced. In mixed blocks, each trial type (response demand) was presented in random order, while in pure blocks, only one trial type was presented, rendering response demand constant for these blocks. It was mandatory to keep the thumbs on the buttons in every block even when no manual response was required. In overlap condition trials, the color-cue remained present the entire trial duration. In gap condition trials, the cue disappeared after 500 ms, yielding a 200 ms gap period between cue offset and target onset. At the end of each trial, the screen turned black for a 1000 ms inter-trial interval before the next trial started.

### Design

Eye movements with an amplitude of at least 4° (halfway) toward the corresponding target location were defined as valid saccades. Error rates (ERs) were analyzed as a function of the within-subject independent variables response demand (single saccade, single manual, dual), fixation condition (overlap, gap), and block type (mixed, pure). Errors included false-negative errors (e.g., absence of a saccade in single saccade trials or dual trials), directional errors (wrong button press, saccade toward wrong direction) and, most crucial for the current study, false-positive errors (e.g., presence of a saccade in single manual trials). Note that in each response demand condition, errors in both response modalities could occur (e.g., single saccade response demand: false-negative saccade, false-positive button press; single manual response demand: false-positive saccade, false-negative button press; dual-response demand: false-negative saccades, false-negative button press). We thus analyzed saccade and manual ERs separately using two three-way repeated measures analyses of variance (ANOVAs).^1^

In addition to overall ERs, we further specifically analyzed false-positive errors in single-action trials (i.e., false-positive saccades in single manual trials and false-positive button presses in single saccade trials) in mixed blocks as a function of previous response demand and, as an exploratory analysis, response direction transition. We again conducted separate ANOVAs for saccade errors and manual errors, respectively. Within-subjects factors were previous demand of currently unwarranted response (present vs. absent) and response direction transition (repetition vs. shift). Note that a previous demand of the currently unwarranted action was present in two trial types (e.g., saccade demand in single saccade trials and dual-action trials). Both trial types were therefore combined in a single factor level (i.e., “previous saccade demand present”).

RTs were analyzed in the same way as ERs, and since only correct trials were included, the variable response demand was reduced to only two levels (single vs. dual). Saccade RT was defined as the time interval between target onset and first valid saccade initiation.

## Results

### Data treatment

The main dependent variable of the current study was the rate of false-positive errors in single-action trials (i.e., false-positive saccade errors in single manual trials and false-positive manual errors in single saccade trials) as an indicator of inhibition failures. However, due to the nature of the employed design, ERs contain three error types altogether (false-positive, false-negative, and directional). Trials containing any type of response error were excluded from subsequent reaction time (RT) analysis. Further, responses executed faster than 50 ms after stimulus onset (1% of trials) as well as RTs deviating more than ± 3 standard deviations (SDs) from the individual cell mean (1.25% of trials) were excluded. In cases of violated sphericity assumptions, Greenhouse–Geisser correction was applied (while uncorrected degrees of freedom are reported).

### Error data

**Fig. 2:**
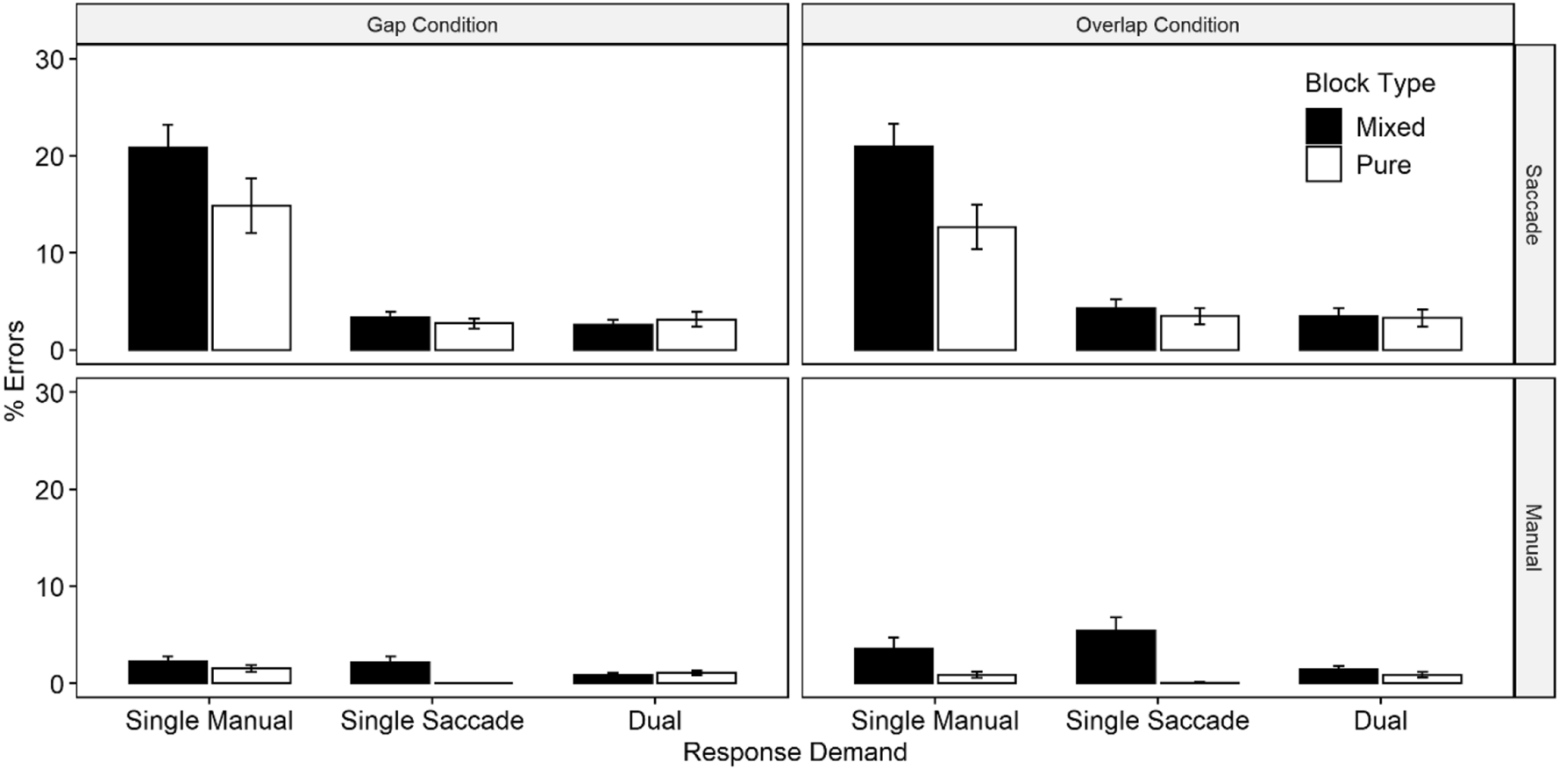
Saccade and manual error rates (%) as a function of response demand, fixation condition, and block type. Error bars represent SE

Overall error data are informative regarding the relative effects of global (action-inherent) and local (transient) differences in action prepotency on inhibition difficulty. Again, global (action-inherent) differences of prepotency should be indexed by the rate of false-positive errors in saccades (compared to manual responses) and in the effect of the fixation condition (gap vs. overlap). Effects of local (transient) changes in action prepotency should be revealed by differences in the false-positive error rates in mixed (compared to pure) blocks. Figure 2 displays mean ERs for saccade and manual responses as a function of response demand (single saccade, single manual, dual), fixation condition (gap, overlap) and block type (mixed, pure). Complete results of statistical tests for saccade and manual responses are referred to in Table 1. In saccade responses, we observed substantial dual-action benefits as reflected in a significant main effect of response demand [*F*(2, 94) = 49.16, *p* < 0.001, ${\eta }_{P}^{2}$ = 0.51]. Bonferroni adjusted post hoc comparisons revealed higher saccade ERs in single manual trials (17.33%) compared with both dual-response (3%) and single saccade trials (3.11%) [both *p*s < 0.001]. Saccade ERs did not differ significantly between single saccade and the dual-response trials [*t* < 1]. There was a main effect of block type [*F*(1, 47) = 9.33, *p* = 0.004, ${\eta }_{P}^{2}$ = 0.17], suggesting fewer overall errors in pure blocks (6.70%) relative to mixed blocks (9.24%). Importantly, these main effects were qualified by a significant interaction of response demand and block type [*F*(2, 94) = 14.36, *p* < 0.001, ${\eta }_{P}^{2}$ = 0.23]. Dual-action benefits were significant in both pure and mixed blocks (*p* < 0.001, *p* < 0.001, respectively), but greater in mixed blocks (18% points) compared with pure blocks (11% points), overall indicating an effect of transient trial-by-trial effects on saccade prepotency. Interestingly, neither the main effects of fixation condition [*F*(1, 47) < 1] nor the interactions of response demand and fixation condition were significant [*F*(2, 94) = 1.08, *p* = 0.255, ${\eta }_{P}^{2}$ = 0.001], suggesting negligible effects of increased global (action-inherent) saccade prepotency in gap (vs. overlap) conditions on the extent of dual-action benefits. We found no other significant effects in saccade ERs.

**Table 1:** Overview of statistical test results based on two separate ANOVAs for saccade and manual error rates

Source of variation	*df*	Saccade			Manual		
		*F*	*p*	${\eta }_{P}^{2}$	*F*	*p*	${\eta }_{P}^{2}$
RD	2, 94	49.16	< 0.001	0.51	4.59	0.044	0.06
FC	1, 47	0.02	0.892	0.00	5.08	0.070	0.07
BT	1, 47	9.33	0.004	0.17	23.01	< 0.001	0.33
RD × FC	2, 94	1.20	0.295	0.03	2.09	0.041	0.07
RD × BT	2, 94	14.36	< 0.001	0.23	13.21	< 0.001	0.24
FC × BT	1, 47	1.27	0.265	0.03	5.65	0.021	0.11
RD × FC × BT	2, 94	0.58	0.536	0.01	2.18	0.123	0.04

Effect size: partial *η*^2^; experimental factors: response demand (RD; single manual vs. single saccade vs. dual), fixation condition (FC; gap vs. overlap), block type (BT; mixed vs. pure)

For manual ERs, the main effect of response demand was also significant [*F*(2, 94) = 3.23, *p* = 0.044, ${\eta }_{P}^{2}$ = 0.06], despite a much lower general level of errors in the manual (vs. oculomotor) domain. Bonferroni-corrected post hoc comparisons revealed that, overall, manual ERs did not differ between response demands (all *p*s > 0.05), indicating, overall, no significant dual-action benefits based on manual inhibition failures. The main effect of block type was significant, too [*F*(1, 47) = 22.16, *p* < 0.001, ${\eta }_{P}^{2}$ = 0.33]. Manual error rates were, overall, lower in pure blocks (0.73%) compared to mixed blocks (2.62%). These main effects were, however, qualified by a significant interaction of action demand and block type [*F*(2, 94) = 14.40, *p* < 0.001, ${\eta }_{P}^{2}$ = 0.24], which was mainly driven by the a greater number of false-positive button presses in single saccade trials in mixed blocks (3.79%) compared with pure blocks (0.03%). Again, the higher frequency of inhibition failures in mixed blocks (vs. pure blocks) corroborates the relevance of transient changes in manual action prepotency. Unlike in saccade ERs, we here found a significant interaction of response demand and fixation condition [*F*(1, 47) = 3.43, *p* = 0.041, ${\eta }_{P}^{2}$ = 0.07] as well as a significant interaction of fixation condition and block type [*F*(2, 94) = 5.71, *p* = 0.021, ${\eta }_{P}^{2}$ = 0.11]. Manual errors were, overall, more frequent in the overlap compared with the gap condition, but only in mixed blocks.

### Sequential effects and further exploratory analyses

In addition to the overall error analysis that clearly indicated an influence of the presence (vs. absence) of local (transient) changes in action prepotency on the frequency of inhibition failures, sequential effects in single-action trials of mixed blocks were analyzed to gage the specific nature of these local (transient) effects. Strong sequential effects in terms of more false-positive errors (in any modality) after a previous executive demand (compared to a previous inhibitory demand) are indicative of local (transient) prepotency based on persisting activation from previous trials. The absence of any strong sequential effects would, in turn, indicate that local (transient) action prepotency was mainly based on the expectation of future executive demands. Furthermore, an exploratory analysis of false-positive errors as a function of previous response demand (executive, inhibitory) and stimulus direction transition (repetition, switch) should inform about whether local (transient) changes of prepotency are driven by representational inertia of the activation of fully specified actions or only modality codes—irrespective of direction.

**Fig. 3:**
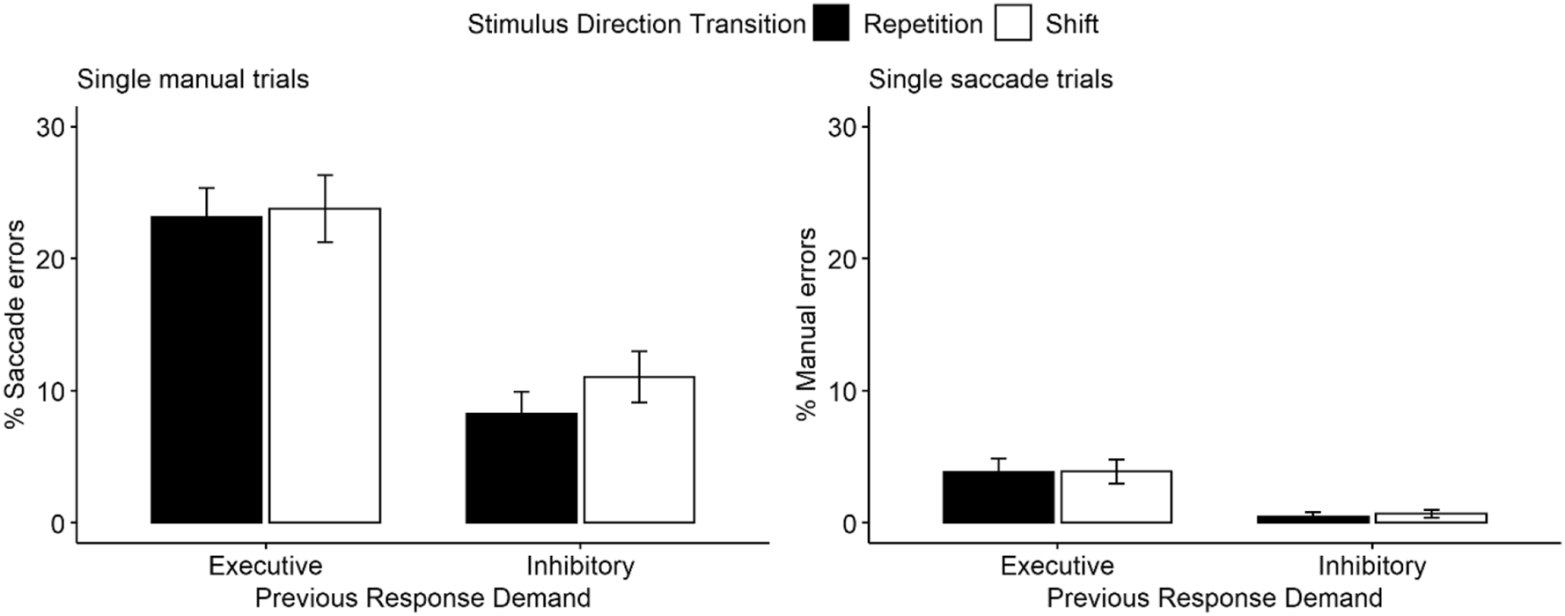
Sequential effects in single-action trials. Saccade errors in single manual trials and manual errors in single saccade trials both as a function of previous response demand and stimulus direction transition. Error bars represent SE

Saccade and manual ERs in single-response trials of mixed blocks as a function of previous response demand and stimulus direction transition are visually depicted in Fig. 3. The analysis of sequential effects indicated that previous response demand significantly affected the rate of false-positive errors in the current trial. For saccade errors in single manual trials, the main effect of previous response demand was significant [*F*(1, 47) = 63.25, *p* < 0.001, ${\eta }_{P}^{2}$ = 0.57]. False-positive saccade errors were significantly more frequent after a previous executive saccade demand (23%) than after a previous inhibitory saccade demand (10%) (*p* < 0.001). The main effect of direction transition was not significant [*F*(1, 47) = 2.17, *p* = 0.147, ${\eta }_{P}^{2}$ = 0.04]: False-positive saccade errors did not differ as a function of stimulus direction transition. The interaction of previous response demand and stimulus direction transition was also not significant (*F* < 1). We found a significant main effect of previous response demand on manual errors in single saccade trials [*F*(1, 47) = 15.73, *p* < 0.001, ${\eta }_{P}^{2}$ = 0.25]. False-positive button presses occurred more frequently after a previous executive manual response demand (3.86%) than after a previous inhibitory manual demand (0.59%). We again found no significant main effect of direction transition and no significant interaction (both *F*s < 1).^2^ Complete statistical results for sequential effects are reported in Table 2.

**Table 2:** Overview of statistical test results based on two separate ANOVAs for sequential effects on false-positive saccade errors in single manual trials and false-positive manual errors in single saccade trials

Source of variation	*df*	Single manual trials			Single saccade trials		
		*F*	*p*	${\eta }_{P}^{2}$	*F*	*p*	${\eta }_{P}^{2}$
PRD	1, 47	63.25	< 0.001	0.57	15.73	< 0.001	0.25
SDT	1, 47	2.17	0.147	0.04	0.04	0.836	0.00
PRD × DT	1, 47	0.84	0.364	0.02	0.02	0.880	0.00

Effect size: partial *η*^2^; experimental factors: previous response demand (PRD; present vs. absent), stimulus direction transition (SDT; repetition vs. shift)

### Reaction time data

**Fig. 4:**
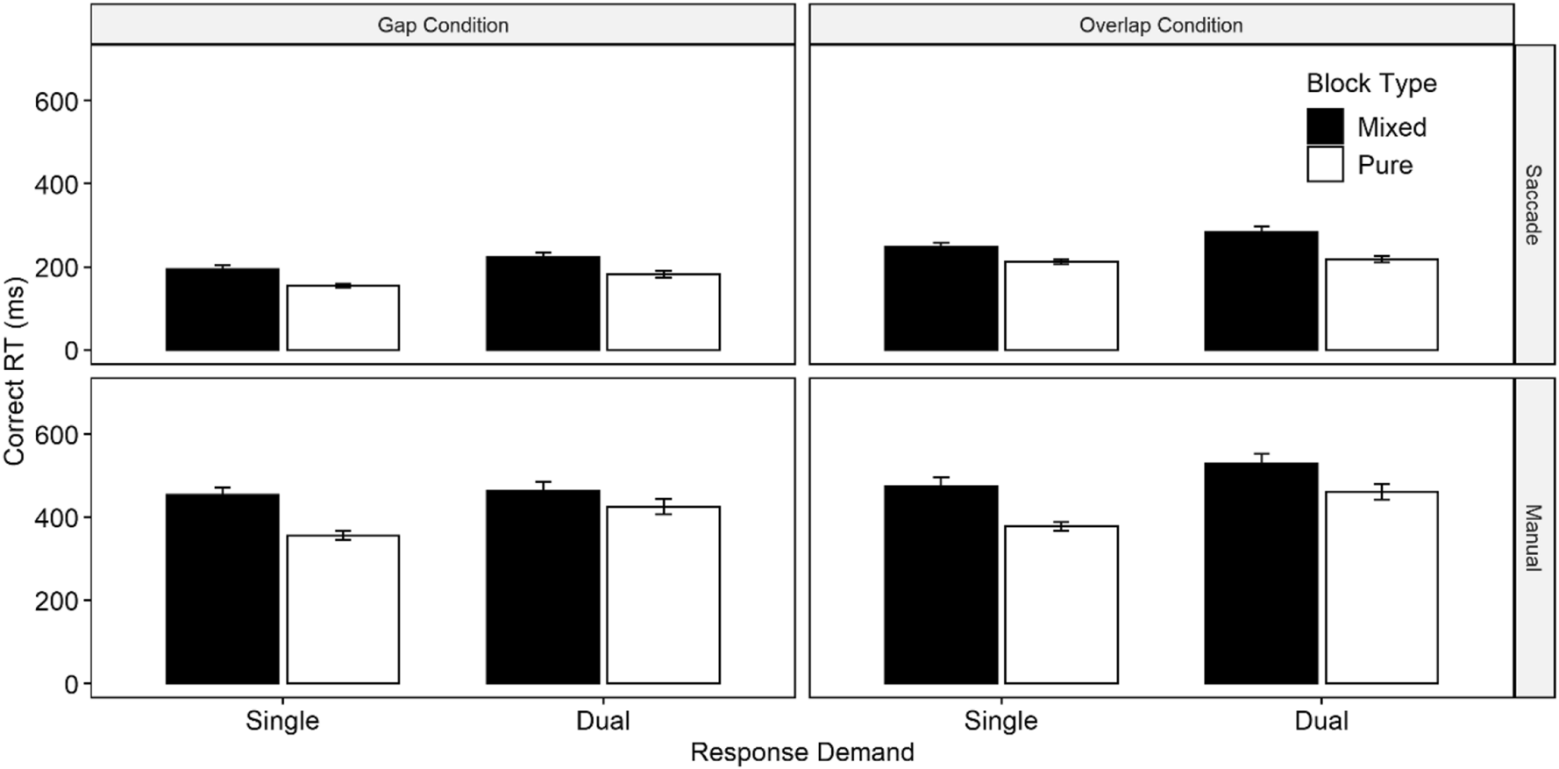
Saccade and manual reaction times (RTs) as a function of response demand, fixation condition, and block type. Error bars represent SE

Complete statistical results for both correct saccade and manual RTs, which were not central to our present research questions, are referred to in Table 3.^3^ Mean correct RTs for both modalities as a function of block type, fixation condition and response demand are visually depicted in Fig. 4. We essentially observed the same pattern of results for both saccade and manual RTs: Responses were overall slightly faster in single-response trials compared with dual-response demands (i.e., indicating overall dual-action costs in RTs). Responses were faster in gap conditions compared with overlap conditions (i.e., indicating a gap effect). Responses were faster in pure blocks compared with mixed blocks (i.e., suggesting mixing costs). Notably, there were also significant interactions with the factor response demand. Specifically, there were no significant dual-action costs in RTs in the overlap condition in pure blocks for saccade RTs (*p* = 0.778), and no significant dual-action costs in gap conditions in mixed blocks for manual RTs (*p* = 1.000). Thus, similar to previous studies, dual-action benefits in error rates were in some (but not all) conditions and response modalities associated with dual-action costs in RTs (see following section for further discussion).

**Table 3:** Overview of statistical test results based on two separate ANOVAs for (a) saccade and manual reaction times (RTs) and (b) saccade and manual error rates

Source of variation	*df*	Saccade			Manual		
		*F*	*p*	${\eta }_{P}^{2}$	*F*	*p*	${\eta }_{P}^{2}$
(a)							
RD	1, 47	27.16	< 0.001	0.37	23.46	< 0.001	0.33
FC	1, 47	142.03	< 0.001	0.75	22.57	< 0.001	0.32
BT	1, 47	27.31	< 0.001	0.37	31.67	< 0.001	0.40
RD × FC	1, 47	1.48	0.230	0.03	6.55	0.014	0.12
RD × BT	1, 47	2.94	0.093	0.06	7.39	0.009	0.14
FC × BT	1, 47	1.32	0.256	0.03	1.54	0.221	0.03
RD × FC × BT	1, 47	7.37	0.009	0.14	4.33	0.043	0.08
(b)							
RD	2, 94	49.16	< 0.001	0.51	4.59	0.044	0.06
FC	1, 47	0.02	0.892	0.00	5.08	0.070	0.07
BT	1, 47	9.33	0.004	0.17	23.01	< 0.001	0.33
RD x FC	2, 94	1.20	0.295	0.03	2.09	0.041	0.07
RD × BT	2, 94	14.36	< 0.001	0.23	13.21	< 0.001	0.24
FC × BT	2, 94	1.27	0.265	0.03	5.65	0.021	0.11
RD × FC × BT	2, 94	0.58	0.536	0.01	2.18	0.123	0.04

Effect size: partial *η*^2^; experimental factors: response demand (RD; single vs. dual), fixation condition (FC; gap vs. overlap), block type (BT; mixed vs. pure)

## Discussion

In the present study, we set out to determine the relative importance of action-inherent (i.e., temporally stable, global) and transient (i.e., dynamically changing, local) action prepotency in the occurrence of dual-action benefits resulting from inhibitory failures in single-action trials. Participants responded to a single peripheral visual target with either a saccade, a manual button press, or both. Global (action-inherent) action prepotency levels were manipulated by contrasting manual action control with oculomotor action control. In previous studies (e.g., Huestegge & Koch, 2014), saccades to peripherally occurring targets have been shown to be particularly difficult to inhibit in a single-/dual-action switch paradigm. In addition, we also manipulated fixation condition (overlap, gap), as the introduction of gap conditions might further boost oculomotor action prepotency. Most importantly, single-action and dual-action demands either varied randomly within a block (i.e., mixed blocks) or were held constant throughout a block (i.e., pure blocks). This allowed us to directly assess the contribution of local (transient) sources of action prepotency (which should selectively play a major role in mixed blocks) to the emergence of inhibitory failures as reflected in the observation of dual-action benefits (see also Los, 1996, 1999).

### Summary of main findings

We found substantial dual-action benefits based on oculomotor inhibition failures in single manual trials and a trend toward dual-action benefits based on manual inhibition failures in single saccade trials.^4^ The marked difference in false-positive error levels between oculomotor and manual control systems clearly highlights that saccades to peripheral targets are characterized by greater global (action-inherent) action prepotency than manual key press responses. Thus, global prepotency, which here is inherent in a particular type of response in a specific effector system, clearly plays a major role in determining inhibitory failures underlying the observation of dual-action benefits. Unlike in the previous study by Huestegge and Koch (2014), where a small but significant effect of fixation condition on inhibitory saccade control was reported, we did not find an additional increase of oculomotor inhibition failures in gap (vs. overlap) conditions, despite a robust gap effect in RTs. In the present setting, the introduction of the gap condition had a slightly different effect, namely on manual ERs, which were less pronounced in gap (vs. overlap) conditions in mixed blocks. One might speculate that gap trials may have resulted in more salient response demands in general (due to the salient offset of the central fixation cross). This could have been particularly useful in the more difficult mixed blocks, where manual response control may thereby have been facilitated.

Most importantly, however, response demand context had a major impact on the frequency of inhibition failures in both modalities. When comparing mixed blocks with pure blocks, false-positive saccades were markedly reduced (but still substantial) in the latter, while false-positive manual button presses were completely absent in pure blocks. These differences between block types indicate that local (transient) action prepotency indeed plays a very important role in determining inhibition difficulty. On the other hand, the persisting dual-action benefits based on false-positive saccades in pure (single manual) blocks demonstrate that a strong global (action-inherent) prepotency of oculomotor responses can still suffice, even in the absence of local (transient) sources of prepotency, to give rise to inhibition failures. The high global (action-inherent) prepotency of the oculomotor actions in the present study indicates that saccades to peripherally occurring targets are executed in a relatively automatic manner (Huestegge et al., 2019).

Furthermore, we found clear effects of local (transient, trial-by-trial) fluctuations in action prepotency on false-positive error rates (see Raettig and Huestegge, 2021 for corresponding effects on RT). In mixed blocks, the rate of false-positive saccade (manual) errors was to a large extent affected by the presence of saccade (manual) response demands in the preceding trial, irrespective of the particular direction of the action in the previous trial (i.e., same vs. different direction). Note that the trial-by-trial effects in false-positive error rates account for the major portion of the block type effect, suggesting that local (transient) effects were nearly exclusively based on persisting activation of response modality activation from one trial to the next, and therefore to a negligible extent due to any anticipation-based mechanisms, such as being prepared for oculomotor responses in an upcoming single saccade or dual-action trial. Taken together, our results therefore show that both global (action-inherent) and local (transient) sources of action prepotency play an important role in determining inhibitory control difficulty.

### Relation to other studies and fields of research

We interpreted false-positive responses in single-action trials as indications of inhibitory control failures. Inhibitory control has long been considered a major executive function (Miyake et al., 2000), and it is typically studied using the stop-signal paradigm (e.g., Logan, 1994; Logan & Cowan, 1984). In this paradigm, an imperative stimulus is (after some time) followed by a stop signal prompting participants to interrupt their initiated response(s). The main dependent variable in this setup is an estimate of the minimum time needed to successfully stop the action, the stop-signal reaction time (SSRT). In the context of the present research, particularly studies on so-called selective stopping (Bissett & Logan, 2014) show some similarity with the current experiment in that one but not another response needs to be stopped. Selectively stopping one response can lead to prolonged RTs in the alternative response (Aron & Verbruggen, 2008; Coxon et al., 2007), which would be comparable to dual-action benefits in RTs in the current study (which we did not observe; but see Raettig and Huestegge, 2021). An important difference in our current design, however, is that we did not implement explicit stop signals after the imperative stimuli. Instead, inhibitory demands in single-action trials were already indicated by the cue 700 ms prior to imperative stimulus onset; thus, participants were not involved in stopping an already initiated response. The same holds for pure blocks. Participants therefore knew in every trial which response to inhibit (and which to execute) prior to the actual response demand. False-positive errors represent only instances in which response inhibition failed in the first place, suggesting different mechanisms in the present study compared with selective stopping paradigms.

Another field of research relevant for the interpretation of the current results is dual-task control. Corresponding theories were traditionally put forward to explain costs of executing two concurrent tasks (typically also involving two actions) in RTs in terms of structural processing bottlenecks that only allow for a single response selection at a time (Pashler, 1994), in terms of the flexible sharing of limited processing capacity (Navon & Miller, 2002; Tombu & Jolicoeur, 2003), or in terms of information crosstalk between simultaneously activated response codes (Hommel, 1998; Navon & Miller, 1987). Originally, none of these frameworks were devised with accuracy-related dual-action benefits in mind. Nevertheless, when conceptualizing a single-action trial in our present study in terms of a dual-task consisting of an executive task combined with an inhibitory task, it appears principally possible to reconcile dual-task theory (in particular, resource sharing or crosstalk accounts, see Huestegge and Koch, 2014 for a discussion) with our present results. More specifically, research on no-go backward crosstalk effects (no-go BCEs; e.g., Miller, 2006) demonstrated that high general go action prepotency in Task 2 can lead to longer RTs in a choice reaction Task 1 in no-go Task 2 trials, representing costs associated with inhibition of another action (Janczyk & Huestegge, 2017; Röttger & Haider, 2017). However, in contrast to the current study, inhibitory demands in no-go BCE settings were imposed by a specific no-go stimulus that needed to be explicitly processed, to some extent similar to a stop signal. In addition, go action prepotency was only varied on a global level by manipulating the ease of go action execution (Janczyk & Huestegge, 2017). Thus, transient effects based on local trial-by-trial sequences were not explicitly considered in this literature (for a notable exception, see Janczyk, 2016).

In contrast, effects of trial sequences are of crucial relevance in the field of task switching. In typical experiments, participants either repeat a task or switch from one task to another (or between different task characteristics, such as response modalities), typically yielding better performance in repetition (vs. switch) trials (for general reviews, see Kiesel et al., 2010; Koch et al., 2018 and Vandierendonck et al., 2010). On a theoretical level, inhibitory control is an important concept to explain such switch costs in this field: It is assumed that in switch trials, a competing task set that was previously relevant needs to be inhibited in a current trial (Koch et al., 2018). However, task switching studies differ from our present setup in that they usually only involve the execution of a single action in each trial. This renders a direct comparison of underlying mechanisms rather difficult. Nevertheless, some task-switching studies focus on performance comparisons between pure blocks and mixed blocks, and also refer to greater working memory demands (similar to our proposed mechanisms behind local, transient effects) as an explanation (e.g., Allport et al., 1994; Jersild, 1927; Spector & Biederman, 1976).

### Theoretical implications and mechanisms

The present data pattern can be readily explained by assuming content (i.e., code-)-based activation patterns within a network of mental representations of relevant task features (including response demands) and their connections during response initiation (e.g., Frings et al., 2020; Hazeltine et al., 2006; Hommel, 2009; Huestegge & Koch, 2010a; Navon & Miller, 1987). When codes of all potentially relevant actions are active to some degree and responses share a common (e.g., spatial) dimension, activation from the currently relevant code binding pattern (e.g., single manual left) might spread to the currently irrelevant action modality code (e.g., saccade). This erroneous activation is more likely, the higher the baseline activity (representing prepotency) of the particular code (e.g., of the oculomotor modality). The present results clearly demonstrate that action prepotency and, in turn, inhibition difficulty depend on both global (action-inherent) and local (transient) factors.

**Fig. 5:**
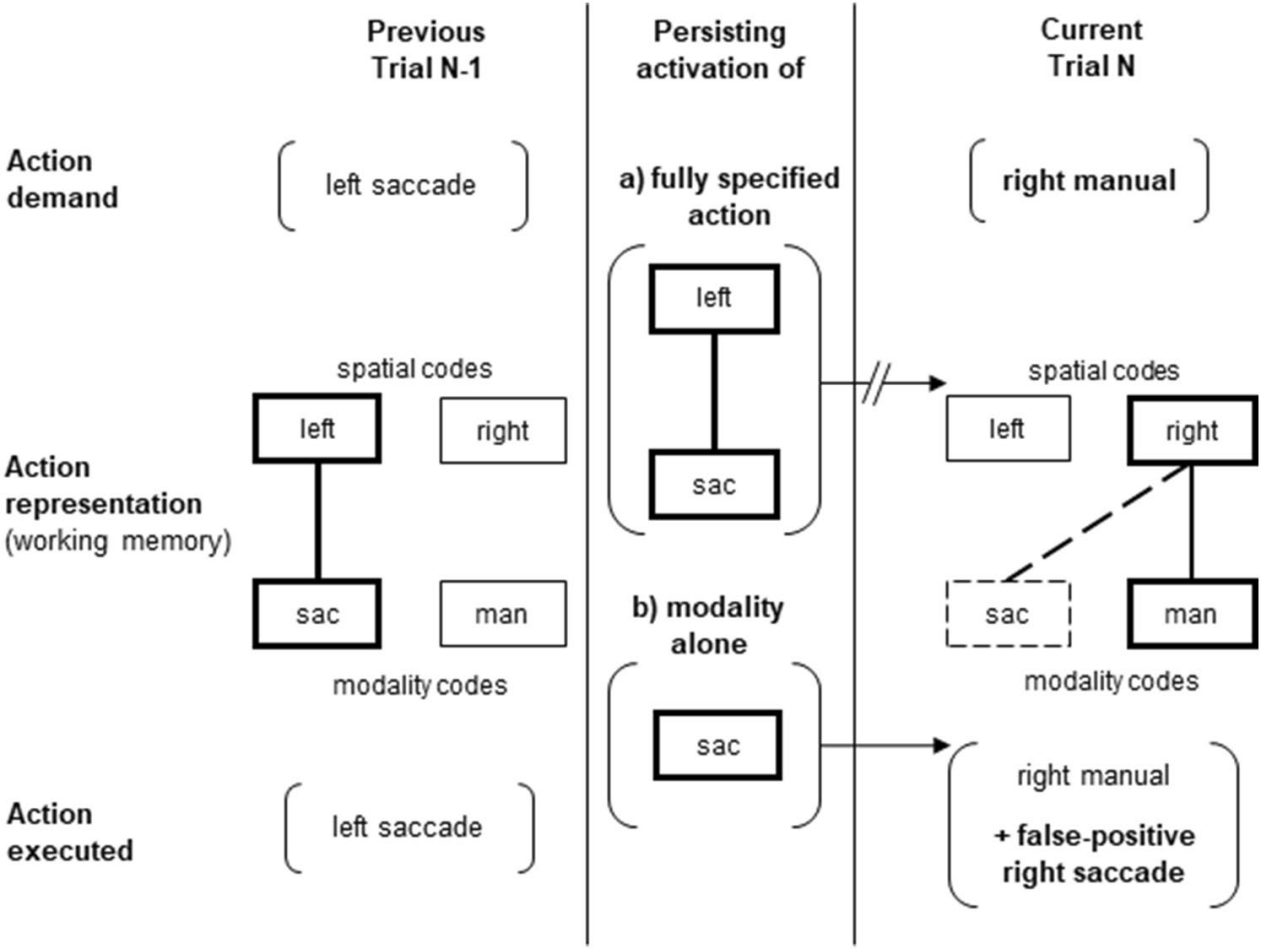
Two potential mechanisms underlying inhibition failures due to local (transient) changes in saccade prepotency. Trial N-1 required a saccade to the left, Trial N requires a right manual button press and *no* saccade. Theoretical assumption a) “persisting activation of the fully specified action from Trial N-1” would only affect prepotency of a saccade to the left in Trial N, thereby *not* increasing the probability of a false-positive saccade to the right. Theoretical assumption b) “persisting activation of the oculomotor modality representation alone” affects saccade prepotency in Trial N irrespective of response direction in Trial N-1, thereby also increasing the probability of a false-positive saccade to the right

Importantly, inhibitory control does not appear to work at the level of fully specified responses, as this should have led to inhibition failures only when the to-be-inhibited action in the current trial has the same directional property as the executed action in the previous trial. Instead, inhibitory control (and its failure) appears to arise on the level of output modality representations in the task set (for similar mechanisms in the context of set-level control in the congruency sequence effect with input modality as a key determinant of task set, see Grant et al., 2020 and Hazeltine et al., 2011). Specifically, a trial involving a particular response modality can be assumed to lead to increased activation of a corresponding response modality representation, which then decays only slowly (or otherwise interferes with the following trial). In this way, it is more likely to be erroneously activated again in the following trial, yielding a corresponding false-positive response (see Fig. 5).

Both global (action-inherent) prepotency differences and local (transient) prepotency changes seem to exert their influence mainly on the level of output modality representations. It should be noted, however, that we do not assume global (action-inherent) prepotency of saccades to be strong enough to trigger substantial inhibition failures in any situation. Instead, it seems more likely that global (action-inherent) prepotency should also depend on the characteristics of a specific task set (including the concrete input modalities and the output modalities of the respective alternative action, see also Pieczykolan and Huestegge (2014) and Stephan et al., (2013)). Thus, the notion of global (action-inherent) prepotency rather refers to a temporally stable basic activation of an action representation in a given task environment. Local (transient) prepotency, on the other hand, refers to fluctuations of this activation based on specific events occurring during the time on task. Consequently, it is reasonable to assume that both sources of prepotency are differentially affected when the input (or the concurrent output) modality changes (e.g., Huestegge & Koch, 2010a, 2013 for examples of settings involving auditory stimuli in which only negligible amounts of false-positive saccade errors were observed in single-manual blocks). Therefore, future studies should further study how exactly input (and concurrent output) modalities modulate both global and local prepotency of different action modalities.

### Implications for applied contexts

The proposed mechanisms of inhibition difficulty in multiple action control settings may also serve as explanatory accounts for some applied phenomena. An example would be the tendency of drivers to steer toward fixated objects (Schneider & Huestegge, 2019). This tendency might be partially rooted in the inability to inhibit a highly compatible steering action when concurrently making a saccade toward a potentially relevant object. Furthermore, false-positive errors also serve as an important diagnostic measure of (in)attention, for example in tasks like the sustained attention to response task (SART, Robertson et al., 1997). Recent findings have demonstrated that instead of inattention due to absentmindedness, false-positive error rates in this type of task might be mainly affected by response strategies based on factors such as the frequency of inhibitory demands (e.g., Wilson et al., 2016). Even though no concurrent action requirements are typically present in the SART, differences in global (action-inherent) and local (transient) prepotency in the respective task context might partially account for some of the false-positive errors committed. Finally, our data also suggest that considerable inter-individual differences exist regarding the ability to suppress unwarranted actions, in particular saccades. This is interesting in light of literature suggesting that anti-saccade performance (which also crucially involves the inhibition of executing a saccade to a peripherally occurring target, see Massen, 2004) is a strong indicator of working memory abilities (Engle et al., 1995; Redick et al., 2007; Roberts et al., 1994), which in turn are closely associated with general intellectual abilities (Kane & Engle, 2002). Therefore, it would be interesting to correlate inhibitory failure liability in our design with assessments of working memory capacity, executive control, and intelligence in future studies to shed further light on the interplay of these concepts.

### Conclusion

Taken together, the present study provides three main contributions to the understanding of accuracy-related dual-action benefits based on inhibitory control failures. First, we demonstrated that besides the impact of different levels in global (action-inherent) prepotency (higher for saccades than for manual actions) on the occurrence of dual-action benefits, there was a particularly strong dependency of dual-action benefits on local (transient) fluctuations in action prepotency, which were present only in a dynamically changing response demand context. Second, regarding these local (transient) effects, the contribution of persisting activation of action modality representations exerted a stronger effect (as evident in the trial-by-trial effects) than anticipation-based processes. Finally, we showed that relatively automatic eye movements, even under most favorable conditions for inhibition (i.e., in a constant response demand context without prior or subsequent oculomotor response demand), are often more difficult to suppress than to execute along with another similar action in a different (here: manual) modality.

## Footnotes

1 In the initial analysis of the manual error data, we noticed an unusually large proportion of false-negative errors when averaging over all participants (i.e., button presses were apparently often omitted in single manual and dual-action trials, irrespective of condition). A subsequent check of the button box utilized to record manual responses revealed a slight insensitivity of the left-hand response button: It did not record a response when pressed too “softly”. This inflated left-hand false-negative error rates in participants with a tendency toward soft button presses (while right-hand false-negative error rates were unaffected). To avoid both an unnecessarily costly re-testing of participants and at the same time any systematic bias in our results, we decided to analyze only right-hand manual error data in those participants with a significant imbalance of left/right false-negative errors (N = 18) as a better proxy for the true proportion of false-negative manual errors in this sub-sample.

2 Note that since sequential effects on error rates were analyzed in single-action trials in mixed blocks only (saccade errors in single manual trials, manual errors in single saccade trials), a maximum of 60 trials was available per participant per modality. Differentiating between stimulus direction repetitions and shifts further reduced this number, potentially reducing the power to detect strong effects. The analysis of stimulus repetitions (vs. shifts) should thus be regarded as exploratory, and the results should be interpreted with some caution.

3 As a further exploratory analysis, we compared error RTs of false-positive saccades (averaged over fixation condition) in single-manual trials of both mixed (*M* = 540 ms, SD = 298.3 ms) and pure blocks (*M* = 751, SD = 429.8 ms) with correct saccade RTs in single saccade and dual-action trials. False-positive saccades were, overall, slower than correct saccades in single saccade and dual-action trials both in mixed and in pure blocks (all *p*s < 0.001). While most of the saccade error RTs were in the same range as correct saccade RTs, a substantial number of false-positive saccades were committed far later than the required manual response.

4 Dual action benefits in saccade accuracy were accompanied by significant dual action costs in saccade RTs. To rule out a speed–accuracy trade-off as a general explanation behind the occurrence of dual-action benefits in ERs we conducted a post hoc analysis of saccade errors for participants without significant dual-action costs in RTs. As a result, the reduced sample (*n* = 25) still exhibited robust dual-action benefits as indicated by a significant main effect of response demand [*F*(1, 47) = 25.15, *p* < 0.001, ${\eta }_{P}^{2}$ = 0.51] This, as well as corresponding findings in previous studies (see Introduction) in which no dual-action costs for saccade RTs were observed, clearly speak against shifts along a speed–accuracy trade-off as the main reason for the present findings.
